# Semi-local Time sensitive Anonymization of Clinical Data

**DOI:** 10.1038/s41597-024-04192-1

**Published:** 2024-12-20

**Authors:** Freimut Gebhard Herbert Hammer, Mateusz Buglowski, André Stollenwerk

**Affiliations:** RWTH Aachen University, Informatik 11 – Embedded Software, 52056 Aachen, Germany

**Keywords:** Medical research, Respiratory signs and symptoms, Experimental models of disease

## Abstract

A method for the anonymization of time-continuous data, which preserves the relation between the time- and value dimension is proposed in this work. The approach protects against linking- and distribution attacks by providing *k*-anonymity and *t*-closeness. Distributions can be generated from given sets using Distribution Clustering, according to the similarity of the curves, which serve as a replacement for the population distribution. Before the data is anonymized, it is split along the time-axis using Windowed Fréchet Splitting, to reduce the duration and information loss. The proposed approach employs bucketization using the Fréchet distance with an implicit maximum cost and implied *t* for closeness and multiple redistribution phases. The information loss, median relative error and achieved *t* for the closeness is low, and the runtime was reduced with the introduction of semi-local decisions.

## Introduction

Data is very important for the progress of science. This is often in a conflict with the sensitivity of the gathered data and the participants’ privacy. Therefore, the data cannot be published “as is” without threatening the participants’ personal rights. This is especially true in a medical environment, where the patients have special rights with respect to their data and the privacy of their treatment. Moreover, measures need to be taken to avoid (re)identification of individuals and other privacy threatening attacks.

Naively, anonymity could be achieved by removing explicit identifiers. But linking attacks^1^ and distribution attacks^2,3^ are only two examples which highlight the need for privacy beyond the removal of explicit identifiers. Intuitively, full privacy would be achieved, if the conclusions of an attacker about an individual are the same, no matter if the individual is actually in the data or not. However, securing the rights of the individuality is always a trade-off between privacy and utility of the data. One has to distinguish between data security, which focuses on protecting the data from unauthorized access, e.g., breaches or data leaks, and privacy, which focuses on ensuring that an individuals sensitive information is protected from unintended disclosure, even under access to the data itself. This paper only focuses on privacy. If security is mentioned, it addresses the achieved level of privacy under the utility - privacy trade-off, which provides protection against attacks on the individuals rights. A full semantic privacy cannot be the goal, as this is not achievable, if the attacker can employ external data sources^4^.

In many fields of research, but especially in a medical environment, the gathered data is inherently time continuous. This comes with many challenges, such that the usual approaches would result in low information quality or even unusable data. First of all, the data needs to be protected against identity disclosure.^5^ The disclosure can be in a direct way via explicit identifiers, like a name or address, which are linked to the data or in an indirect way via so called quasi-identifiers, e.g., the combination of weight, height and zip code, which can be combined to identify an individual in the table. At least with the onset of wearable devices, identification of user’s with ECG-based authentication emerged, which may pose a threat to the user’s privacy^6,7^. There is a possible attack on the user’s identity when their ECG time-series data is published, e.g., for scientific reasons, and the adversary may already possess the ECG data from an aforementioned wearable device.

Hence, anonymizing data before publishing is a means to protect the privacy of a patient or user. There are three general approaches to anonymity^4^:

1. perturbation,
2. subsampling and
3. generalization.

A **perturbation** based approach introduces noise to the dataset in order to gain privacy^8^, thus the data truth is not preserved, as data points are disturbed or might even be removed or added^9^.** Subsampling**, i.e., releasing only a random subset of the data is often used as a privacy enhancing mechanism together with other mechanisms^10,11^. It has similar problems as do perturbation-based approaches, as e.g., the data truth is not fully preserved. While no data is added or perturbed by subsampling alone, not the full set, i.e., not the full truth, is released. As it does not protect the individuals which got picked into the subset for release, it can only be seen as a privacy enhancing mechanism only when used together with another mechanism. Specially, when focusing on time-series data, sub-sampling would need to release an entire time-series of a patient or take them fully out. When working with the dynamics of a time-series data-set e.g., analyzing breathing data, where you need to put the course of a single breaths flow and pressure data in relationship, sub-sampling would result in useless data. In contrast to this, a **generalization** based approach uses multiple data points or curves and uses the commonalities of all members to generate a common representation^1^. This can be a value range, e.g., for age, which could generalize three data points {18, 30, 31} to {18–31}. Another possibility is a domain specific non-numeric generalization e.g., for profession, which could generalize {*pediatrician*, *cardiologist*, *internist*} to {*medical* *doctor*}, which could be further generalized by merging {*medical* *doctor*, *nurse*} to {*medical* *professional*}.

The most common and the one used for the presented work is *k*-anonymity, which is a generalization approach. *k*-anonymity provides protection against linking attacks by grouping together *k* data entries and generalizing them^1^. As *k*-anonymity does not protect against distribution attacks further protection is needed. By performing a distribution attack, an adversary gains knowledge about its victim, without re-identifying it. This is due to a large distance between the distribution of sensitive attributes in the equivalence class and the population. E.g., if most members of a victim’s equivalence class have a relatively uncommon disease, the attacker gains the knowledge, that the likelihood of the victim having this rare disease is higher than in the overall population.

Protection against such an attack can be achieved via *t*-closeness, introduced by Li *et al*.^2^. In addition, the assignment of the data to an equivalence class is restricted in such a way that the distribution of the sensitive attributes in the equivalence class is at most *t* apart from the distribution in the population^2^. This ensures diversity where it is needed, while allowing homogenous equivalence classes, where this represents the population. Thus *l*-diversity introduced by Machanvajjhala *et al*.^10^ is not needed.

In order to measure the distance between distributions a suitable metric need to be found. For the approach proposed in this paper the Earth Mover’s Distance (EMD), with an application specific ground distance is used^2,10,12,13^. It measures the amount of work needed to transform one distribution into another. For this the moving of a pile of earth is the usual analogy. I.e., both distributions are seen as hills and valleys, the amount of earth needed from the hills to fill the valleys of the other distribution is taken as the mass. This is then multiplied by the distance between the hill and the valley. This distance is application specific and quantifies the difference of the measured values. Retroactively adding the *t*-closeness constraint on to existing *k*-anonymized equivalence classes is inherently suboptimal, both with respect to information loss and computational effort^12^.

As *k*-anonymity alone is not sufficient to protect against distribution attacks^2,10^, a *t*-closeness centered approach is needed. Such an approach is SABRE, introduced by Cao *et al*.^12^. It is based on a bucketization phase, which allows the merging of all created equivalence classes, while maintaining *t*-closeness. The used mechanism of Cao *et al*.^12^, can be broken down into a bucketization phase, which groups together values based on similarity and a redistribution phase, which distributes the values into equivalence classes based on the distribution of the population in order to achieve *t*-closeness. The equivalence classes are created by recursively splitting the data, while obeying the proportionality constraint w.r.t. the elements an equivalence class should obtain from each bucket. This results in a maximum error and a tree structure of equivalence classes, which can be merged bottom up, thus increasing *k*, while still obeying the proportionality constraint and thus achieving *t*-closeness.

SABRE in contrast, as well as most other existing approaches examined in this paper, lacks the capabilities to handle continuous time-axis such that the data remains usable. This is due to the fact, that they work purely point based, i.e., on a local decision and do not distinguish between the axes. Thus, SABRE could group together points along the time dimension, to achieve a lower information loss. For medical data like an ECG this would render it almost useless despite being a theoretically better grouping. An example for anonymization by perturbation is the work by Bonomi *et al*.^14^, in which a procedure is described to reduce the uniqueness of shared ECG time-series data while preserving its usability for researchers.

While this approach is able to preserve one individual user’s privacy when the domain size is sufficiently large, our focus was on anonymizing a set of waveform data from a group of subjects that show a common phenomenon. Grouping together whole curves, like proposed by Nergiz *et al*.^15^ can become unfeasible for high density, long interval data, which is common in a medical environment. As global decisions on such data are often too expensive and local decisions can lead to a high information loss. Therefore, a *semi-local* decision is proposed in this work. I.e., the data is split into blocks according to a heuristic, addressing the local aspects, and grouping the curves into equivalence classes using a global decider within those blocks, which results in a semi-local approach. The grouping is done similarly to SABRE, but with a time sensitive adaptation.

The generous temporal aggregation of multiple measured values of a continuous data stream can be a naive way of anonymization. However, in the case of medical data, in which physiological processes such as breaths or heartbeats are usually repeated periodically, the temporal aggregation of measured values from multiple of these events to a single point in time would result in the data no longer being usable. Therefore, anonymization must also be time-sensitive.

Our presented method takes in a set of waveform data, ideally of equal length and with identical time resolution, and creates anonymized equivalence classes of curves which fulfill the given *k*-anonymity, minimize the information loss within the equivalence classes, and generate a hull as a representative of the original data of that class. We evaluate the method on data acquired during an animal experiment^16^.

## Metrics

In order to anonymize time-continuous data via bucketization, multiple metrics are needed:

1. to measure similarity between curves, in order to be able to group them into buckets and generate a distribution clustering,
2. for the measurement of the distance between distributions, to achieve *t*-closeness and
3. to quantify the information loss, in order to optimize the equivalence class creation.

The similarity metric should not be purely point based. E.g., a naive approach of a pairwise Euclidean distance of the sampled curves would heavily depend on the alignment and shift of the curves, but would not classify similarly shaped curves as similar. An approach based on set of points, like the Hausdorff-distance, can lead to very distinct curves being judged as similar, as was discussed by Alt and Godau^17^. The Fréchet-distance on the other hand takes the resemblance into account when calculating the distance between two curves. An intuition for this distance metric, which was introduced by Eiter and Heikki^18^, can be a dog leash. There is a dog and its owner, they are connected with the leash. The dog walks on one curve, the owner on the other. Both can walk forward or stop for any time, but never walk backwards on the curve. In this scenario the Fréchet distance is the minimal length of the leash, such that both dog and owner reach the end of their curve. For all aforementioned three applications the ground distance of the Fréchet distance is the Euclidean distance. We chose the Fréchet distance as the similarity metric between any two curves, thus allowing the clustering of curves in the approach proposed in this paper. The approach taken by Dynamic Time Warping (DTW) does also allow comparing the similarity of two curves and is less sensitive to outliers due to the summation of the costs rather than minimizing the maximum distance. Yet, DTW holds two undesired properties for the proposed methodology in this work. DTW is not a metric as it does not fulfill the triangle inequality, thus rendering DTW insufficient to compare multiple curves.^19^ Furthermore, where the course over time and the time itself are important features of the curves, DTW makes two similar shaped but different length curves seem close together. In our worked example using breath signals, the respiration rate, and thus the length of the breath, may be such important features. The Fréchet distance is not only employed as the similarity metric for the clustering into equivalence classes and the creation of distributions, but also as the ground distance for the EMD.

The implemented bucketization addresses the need to conserve the dynamics of the curves. This enables especially the application of the proposed method in the medical domain with clinical data without the direct need to have additional models available, which would be the case if differential privacy would be implemented^20^. Additional physiological knowledge can be utilized by the application of according splitting techniques for the incoming data, which we also show exemplary in the evaluation.

To monitor the performance of our approach, we chose an information loss metric for which there are several candidates. E.g., the classification^21^ or discernibility metric^22^. Both depend on the application specific data usage and are therefore not well suited for the framework developed in this paper. Thus, a different approach, called General Loss Metric (GLM)^21^, is used. This metric is also used for the implementation of SABRE and CASTLE by Cao *et al*.^9,12,23^. The GLM can be seen as the fraction of the domain, spanned by the generalization. I.e., the range of the equivalence class normalized by the range of the domain, for numerical attributes. It is the number of leaves of the Domain Generalization Hierarchy^12^ in the equivalence class, normalized by the number of total leaves, or 0 if there is only one leaf in the equivalence class. This metric provides the means to measure the loss of information for each equivalence class and a whole anonymization of a dataset, i.e., a generalized partition. As the information loss is calculated on the generalized domain alone, the implementation described in this paper works on the hull of the equivalence class when calculating the GLM, rather than every member of it. This can reduce the complexity for equivalence classes with many members, as the information loss has to be calculated for every possible member that might be added.

In order to achieve *t*-closeness, the distance between distributions must be measured^2^. We chose the EMD, with the Fréchet-distance as a ground distance. The sum of all flows between the distributions multiplied by the ground distance for the attribute is the work required to equalize the distributions. The work, normalized by the total flow is the EMD as defined by Rubner *et al*.^13^. This means the difference of observations between the distributions – indicated by the flow – (“the earth”) which need to be moved, multiplied by some ground distance (“the distance the earth is moved”) and normalized by the total flow.

## Preliminaries

**Fig. 1:**
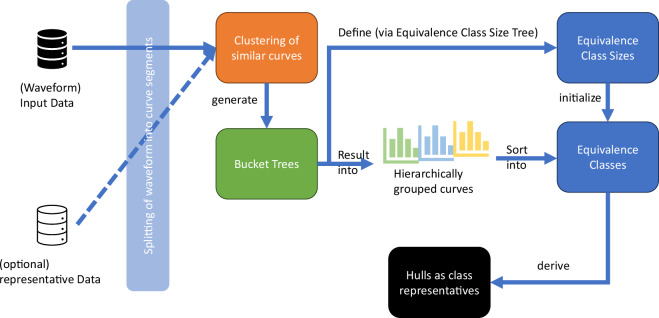
Workflow for the creation of a hull to represent waveform datasets with a desired *t*-closeness and *k*-anonymity.

In this section all concepts are explained, which are needed for the developed anonymization method. The process is additionally depicted in Fig. 1. The process begins with the clustering of the distribution in Distribution Clustering, which preserves the relevant properties of the underlying measurement series in a time-sensitive manner. Subsequently, trees are generated, which are used to group data hierarchically together for the later distribution processes. We call them *Bucket Trees*. Followed by the Equivalence class size trees, which use some results from the Bucket trees and generate, still empty, Equivalence classes, which fulfill the desired *t*-closeness once they are filled with the data from the Bucket tree. Both tree structures are described in Anonymization Trees. Afterward the splitting of the data is explained in Splitting.

### Distribution Clustering

In order to determine the *t*-closeness of the anonymization, a distribution in the population and in each equivalence class is needed. For point based numerical or categorical data this can be done rather intuitively. In the case of time-series data this becomes a challenge. Without clustering of a dataset of such time-series data, the population distribution would be a uniform distribution, with every value having one observation. Therefore, the data is grouped into clusters, with respect to their similarity. A cluster is a set of similar curves, identified by a reference value. The reference value can be any of the members. In the implementation described in this paper, it is the first member added to a cluster. Members are added to a cluster according to a given threshold for the distance of curves: the distribution sensitivity *s*_*d*_. Which means, all curves that are at most *s*_*d*_ apart from the reference key are grouped into the cluster. For a cluster with *n* members, the reference key *r* has *n* occurrences in the distribution. If a distribution is not generated on larger or separate data, i.e., a global distribution, but on the same dataset which will be anonymized, a cluster of the distribution contains only a single element, this will lead to problems in the redistribution from the buckets. As there is only one element in the cluster, either all equivalence classes need to be merged, or the resulting *t* has to be large. This rarely represents the underlying population distribution. Depending on the chosen *s*_*d*_ and the data, clusters with single elements might still occur. They should be eliminated by merging them with the nearest cluster.

The data for the distribution can either be the same as the one to be anonymized, or from a larger dataset. In either case the quality of the resulting distribution has a large impact on the anonymization. If *s*_*d*_ is chosen too large, and there is only one cluster in the distribution, 0-closeness is trivially achieved for any anonymization. This would most likely not represent the real distribution. If *s*_*d*_ is chosen too small, a distribution with many small clusters will result in a high *t* or large *k* and possibly higher information loss for the equivalence classes. Hence, a good, data-specific balance for the distribution sensitivity must be determined by the user.

### Anonymization Trees

The approach, which was developed in this paper relies, similar to SABRE by Cao *et al*.^12^, on bucketizing the data. Before redistributing it proportional to the sizes of the buckets into the pre-calculated equivalence classes the data is divided into the buckets according to similarity. Rather than choosing a maximum cost and closeness *t* for the generation of the buckets explicitly, Bucket trees are introduced. The Equivalence Class Size Trees from SABRE provide the initial target sizes for the equivalence classes. Both types of trees can be summarized as Anonymization Trees.

#### Bucket Trees

A Bucket Tree is used to select a set of buckets for the redistribution of the data into equivalence classes, without choosing the maximum cost explicitly. This ensures that a partition for the chosen cost and thus an anonymization with those constraints is in fact possible.

##### Definition 1

 (Bucket Tree). A Bucket Tree is a fully balanced binary tree, where the maximum difference between the depth of any two leaves is 0. Each node *b*_*i*_ has a value ${n}_{i}\in {{\mathbb{N}}}_{ > 0}$, such that if *b*_0_ is the parent node of *b*_1_ and *b*_2_, *n*_0_ = *n*_1_ + *n*_2_. Every node represents a bucket for the data distribution. Hence, every level in the Bucket Tree provides a set of buckets partitioning the dataset.

According to Definition 1 the sum of values on each level in the Bucket Tree is the number of data entries in the dataset the Bucket Tree is generated from.

The Bucket Tree provides a structure to select sets of buckets, partitioning the data. The partition gets finer with an increasing depth in the Bucket Tree. A value taken from a bucket and added to an equivalence class is treated as the reference key of the bucket, which it is most likely not. Thus, an error is introduced into the distribution of the equivalence class, which must be accounted for. This is done via a maximum cost approximation for each bucket. The maximum cost of a bucket is over-approximated by assuming that every value taken from the bucket is the member with the largest distance to the reference key. The distance is calculated using the Fréchet distance on the common time frame of the data, thus ignoring alignment issues. The maximum cost of a bucket is this distance. The maximum cost of a set of buckets is the maximum of all bucket costs. The calculation of this approximation for the bucket error was introduced for SABRE by Cao *et al*.^12^. It also provides the means to estimate a lower bound for the achievable *t*. It can be adapted by adjusting the grouping of the data and therefore the content of the buckets.

A Bucket Tree provides the user with a tool to select a bucketization, based on a balancing factor for the tree and a depth, rather than an explicit maximum cost. This is done as the manual selection of the maximum cost depends heavily on the similarity and distribution of the similarity of the underlying data. Thus, an a priori estimation of the impact is hardly possible for those parameters.

To create a Bucket Tree, all data is put into one root-bucket. Then all buckets are split recursively with respect to the cost, i.e., information loss. The user then selects a depth in the Bucket Tree and all nodes of the given depth are used in the anonymization. A depth within the Bucket Tree is chosen and all nodes of that depth are used in the anonymization. This approach needs a node for every branch of the tree on the given level.

Therefore, the maximum depth is the minimum depth of all branches. Otherwise, the tree is not fully balanced and, thus, does not partition the dataset on every level, which is a necessary condition of a Bucket Tree according to Definition 1.

**Fig. 2:**
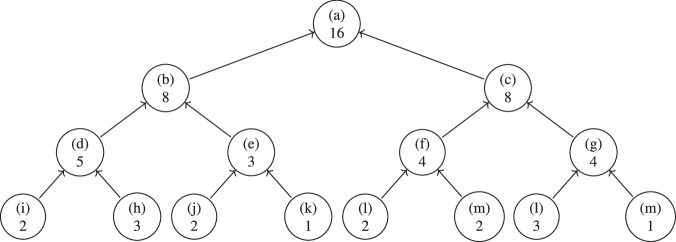
Example – Bucket Tree.

The Bucket Tree in Fig. 2 is created from 16 data entries in the root (a). They are split into two equally sized nodes (b) and (c) with eight entries each. (b) is then split into two smaller nodes (d) and (e) with five and three entries. (c) is split into two equally sized nodes (f) and (g) with four entries each. (d), (e), (f) and (g) can be split further, but after the split of (e), one child node (k) is of size one, thus no further split is possible for the level.

In order to increase the minimum depth, a balancing factor is introduced, i.e., the optimal split is adapted in order to decrease the imbalance in the Bucket Tree, resulting in a deeper tree and thus finer bucketization. Enforcing a perfect balance is not desirable, as this results in equally large buckets for every level, which rarely fits the underlying structure of the data. An overly restrictive balancing factor can result in an overly large maximum cost and thus a bad anonymization. The depth, which implies the number of buckets, should be chosen with the distribution in mind. On the one hand, a low depth, i.e., few buckets, can hardly match a distribution with many clusters. On the other hand, a high depth, i.e., many buckets, may result in a higher computational cost and information loss, with no gain for the result of the anonymization.

#### Equivalence Class Size Trees

The Equivalence Class Size (ECS) Trees were introduced by Cao *et al*.^12^ for the use in SABRE. It is a fully balanced binary tree which is generated by recursively splitting the dataset while maintaining the proportionality to the population distribution. Each node of the ECS tree is a set of virtual buckets that represents an equivalence class and consists of a tuple of *n*_*b*_ elements, where *n*_*b*_ is the number of buckets. The value of each element of the tuple gives the number of elements that shall be chosen from the respective bucket to fill that equivalence class instead of the specific time-series data. Every level of the ECS tree is a complete partition of the dataset. The splitting of the ECS tree stops when there is a virtual bucket with only one element left in at least one bucket.

In contrast to their use in SABRE, the target sizes are only a rough approximation, as a redistribution or splitting of an equivalence class can occur in the approach developed in the present work in order to minimize the information loss. Those virtual buckets are then split, until at least one virtual bucket has only size one and thus cannot be split further. This provides the target size for each equivalence class and the rough number of elements of each bucket it should contain after the anonymization. Furthermore, the parenthood relation of the nodes is a structure for merging the equivalence classes, while maintaining the *t*-closeness.

### Splitting

The information quality can be improved, i.e., the information loss reduced, by dividing the data into smaller regions along the time axis, on which the whole anonymization process is executed respectively. Each part is called a segment. The computational time is not only split, thus allowing a segmental parallelization of the anonymization, but may also be reduced. Furthermore, the curves can change equivalence classes between segments, which can lead to a better set of equivalence classes with respect to the information loss, but at the cost of impeding one continuous hull for any equivalence class over the whole time-range.

Given a curve *c* and the equivalence classes *E*_1_, …, *E*_*n*_ for the non-segmented case, the information loss of adding *c* to the best equivalence class, i.e., the one with the lowest maximum cost of adding *c*, is *l*_*u*_. If there are some regions of the curve *c* where the information loss is very high, it might be better to assign *c* to a different equivalence class for just those regions, to lower the overall information loss. This can be achieved by splitting the curve into curve segments *c*_1_, …, *c*_*m*_ and creating the equivalence classes ${E}_{{1}_{1}},\ldots ,{E}_{{1}_{{n}_{1}}},\ldots ,{E}_{{m}_{1}}\ldots {E}_{{m}_{{n}_{m}}}$. There are *m* segments with *n*_*i*_ equivalence classes each, where *i* is the index of the segment. If the segments match the regions where the local information loss is high, assigning the curve segments locally can improve the overall result. Let *l*_*i*_ be the average information loss of segment *i*. The average segmented information loss of the split dataset $\frac{{\sum }_{i=1}^{m}{l}_{i}}{m}$ can be lower than *l*_*u*_. If the globally optimal solution is also optimal for each segment and every curve, the result will be the same as if the data was not split. This also reduces the computational complexity, as the Fréchet distance is calculated multiple times during the creation of an equivalence class. The complexity of it is $O(p\cdot q\cdot \log (p\cdot q))$, where *p* and *q* are the number of edges for the curves^17^. Thus dividing the curves in *m* segments reduces the complexity for every calculation to $O\left(\left(\frac{p}{m}\cdot \frac{q}{m}\cdot \log \left(\frac{p}{m}\cdot \frac{q}{m}\right)\right)\cdot m\right)$, assuming an equal resolution of the curves over time.

There are three modes to split the data in the proposed framework.

1. The fixed-length approach, which is straight forward. The user enters the length of segment, and the data is split into equally long parts. If there is remaining data at the end which length is lower than a predefined threshold, it is added to the last segment. This approach has almost no effect on the information quality, as it does not take the structure of underlying data into account when performing the splits. But it reduces the runtime of the anonymization.
2. Visually tool-based approach, which allows an expert to select each segment. Every segment has to start after the previous, but data can be omitted between segments. This results in a possibly well segmented data. The splitting takes roughly the same computational effort as the fixed length approach, but needs interaction with an expert.
3. The approach developed in this paper uses the Fréchet distances as a measure of similarity and utilizes a sliding window approach to detect regions of similarity for splitting. The user provides a window, step size and number of segments. Afterward the Fréchet distance is calculated between each curve for every window of all possible splittings and the mean distance is stored. After the whole dataset was processed, the data is split at the position of the windows with the lowest mean distance as beginning of a new segment. Which means that the created segments reach from one region of similarity to another. This results in a varying length of the segments on the one hand but partitioning the whole dataset without gaps on the other hand. With a well-chosen set of parameters, this can provide large segments, in regions with low dynamics and the curves are very heterogeneous, and small segments in those, where more frequent changes occur and the curves are locally very homogeneous.

## Methods

The anonymization approach proposed in this paper is a multiphase process on curves, that comprises the following five phases:

1. Split the data (optional)
2. Load separate population-distribution data (optional)
3. Calculate a distribution
4. Generate a Bucket tree
5. Generate the equivalence classes

A curve is composed of an ordered list of data points {(*t*_1_, *v*_1_), …, (*t*_*n*_, *v*_*n*_)} where *t* is the time since the start of the curve and *s*_*d*_ is the measured value. In general, sufficiently good data quality is the most important precondition for any data handling^24^. Given the broad field of data quality assessment, a deeper discussion how to assess and achieve the quality for the given data is omitted here. Instead, following assumptions are expected to be met by the dataset to be anonymized:

- the data is of good quality, especially all data points are valid
- the dataset consists of approximately equally long samples
- all samples start at the same timestamp

Of course, any dataset of curves may be anonymized with the presented method, but data inconsistencies and outliers may result in non-optimal information loss of the results.

Some preparations need to be done before creating the anonymized equivalence classes, which are briefly described in Preprocessing and Preparations and cover steps 1 to 4. The distribution of the data into equivalence classes is described in detail in Equivalence Class Creation. Anonymization constraints and security aspects are discussed in Privacy Properties.

### Preprocessing and Preparations

The data may be split into segments, as was proposed in Splitting, reducing the otherwise global to a semi-local decision, or the curves are anonymized as they are. The segments for the semi-local approach are generated by splitting each curve. It is called semi-local, as the assignment to the equivalence class is done by a global decision within the segment, not based on the individual points of the curves. If the splitting is omitted, the anonymization is based on a global decision. I.e., the curves must be anonymized across the whole length. E.g., multiple breaths must be matched and anonymized as a whole with other curves containing multiple breaths as well.

There is no further data augmenting or altering preprocessing employed to avoid that distinctive features of equivalence classes get diluted or filtered by removing supposed outliers. By employing the Fréchet distance as the similarity metric, the metric itself is also influenced by those features. Thus, data quality and plausibility are expected to be ensured by the user in advance.

The pseudocode in Algorithm 1 describes the preparations which reflect steps 2 to 4 for each segment. First *distances* are computed as the Fréchet distances over the given curves. For *n* curves, it is a *n* × *n* matrix containing non-negative real numbers. Together with the distribution sensitivity *s*_*d*_, *distribution* is calculated by clustering as the distribution over the global population, as introduced in Distribution Clustering, to compare the equivalence class to. I.e., the global population contains all data available and is not only the data which will be anonymized. If the data to be anonymized is different to the one used for the distribution generation (e.g., a subset of the global population), *distances* is calculated again on the curves, which will actually be anonymized. Thereafter, the Bucket- and Equivalence Class Size Trees are generated, as defined in Anonymization Trees. The curves are grouped into similarity buckets, where every member is treated as if it were the same and are associated with a maximum error, allowing the over approximation of the lowest possible *t*. *distances* is used to generate the Bucket Tree *bTree*, according to the balancing factor *f*. *bTree* provides the buckets for the Equivalence Class Size Tree *targetSizes*, according to the chosen Bucket Tree depth *depth*_*b*_. The target size of an equivalence class is used to determine the number of curves taken from each bucket on each round of the data distribution in order to maintain the proportions of the population distribution and thus *t*-closeness. The buckets of a chosen depth in the trees are later redistributed into the equivalence classes with respect to the other parameters and constraints. Finally, the initial empty equivalence classes *eqcs* are created with initial sizes given by *targetSizes*.

### Equivalence Class Creation

The pseudocode in Algorithm 2 describes the general approach to distribute the data from the buckets into equivalence classes, such that they fulfill *k*-anonymity and *t*-closeness, while maintaining a low information loss. First the initial (empty) equivalence classes, which are generated with their target sizes according to the Equivalence Class Size Tree, are inserted into the *unmarked* queue in Line 1. Afterward in Line 4 data from the buckets is distributed into the prepared equivalence classes, created with the target sizes of the Equivalence Class Size Tree, until all equivalence classes grant the anonymization criteria, or no data remains in the buckets. The *unmarked* queue holds all equivalence classes, which do not yet fulfill the constraints of the anonymization.

If not, all equivalence classes could be anonymized in the first phase, the remaining content of *unmarked* is redistributed between other members of *unmarked*, to create additional anonymized equivalence classes. This phase in Line 7 is called the Unmarked Redistribution phase.

If afterward there are still equivalence classes left in *unmarked*, which do not yet fulfill the constraints of the anonymization, they are redistributed into the already anonymized equivalence classes, which are stored in the *marked* queue. This phase in Line 10 is called Constrained Marked Redistribution, as there are still strict constraints w.r.t. information loss in place for the redistribution.

If even this Redistribution phase does not succeed in anonymizing all data, those constraints are loosened for the Relaxed Marked Redistribution phase in Line 13. In this last phase equivalence classes will no longer be split, but the drop threshold *d* is still in place.

#### Data Distribution Phase

With all prerequisites for anonymizing time-continuous data fulfilled, the first phase in Algorithm 2 is the initial distribution of all curves into the final equivalence classes in Line 4. The data distribution is shown in detail in Algorithm 3. The function *dataDistribution* takes the minimal equivalence class size *k*, the equivalence class sensitivity *s*_*eqc*_, which gives an information loss threshold for how fast an initial equivalence class is split and the drop threshold *d*, which is the upper bound of the information loss, as parameters. If the information loss of the best matching equivalence class for a curve is increased above this threshold, the curve is dropped from the dataset instead of being used, to avoid creating too wide equivalence classes. Dropped means, the data is added to the equivalence class, as it is the best option available, but not used for any further calculations on the equivalence class. For the sake of transparency, every dropped curve can be visualized in the evaluation of the anonymization. Additionally, the population distribution *distrib* and the *buckets* of the chosen depth in the Bucket Tree are used, together with the target size of the equivalence class, to determine the number of elements which have to be taken from each bucket to maintain *t*-closeness for every equivalence class.

The data distribution phase begins by taking an equivalence class *e*, which is empty at the beginning, from the *unmarked* queue and fills it with the contents of the buckets, while obeying the maximum cost constraint, as defined by Cao *et al*.^12^. Constraints on *k*-anonymity and *t*-closeness are given with the equivalence class. The filling is done proportionally w.r.t. the target size of the equivalence class, the total number of elements taken, and the population distribution for the number of elements taken from each bucket. If the information loss is not above the drop threshold, but higher than *s*_*eqc*_, the equivalence class with its current members and target size is split. As soon as the equivalence class fulfills all constraints, it is marked and added to the list of finished equivalence classes *marked*. Unless all equivalence classes are marked, or all buckets are empty a new equivalence class is taken from the *unmarked* queue and the process repeats.

#### Redistribution Phases

A redistribution between the filled equivalence classes begins if not all equivalence classes are marked, i.e., *unmarked* still contains elements and those do not fulfill all constraints. The constraints are *k*-anonymity with *t*-closeness and a maximum cost constraint for the information loss. There are three redistribution phases, first the Unmarked Redistribution Phase in Algorithm 4. It redistributes the content of an unmarked equivalence class into other unmarked equivalence classes. This is done until either all equivalence classes are marked, or no data was added to any equivalence class for a whole iteration, as the maximum cost constraint is still in place.

The next two phases both use the same function *markedRedistribution*() in Algorithm 5, with differing *constrain* flag set to either *CONSTRAINED* or *RELAXED*, respectively. Both take an equivalence class from the *unmarked* queue and fill its content into the member of the *marked* queue. In the Constraint Marked Redistribution Phase the maximum cost constraint for the filling of equivalence classes is still in place, whereas it is omitted in the relaxed variant of the phase. The redistribution is done until the unmarked queue is empty, or no data was added to any equivalence class. However, the latter can never happen for the Relaxed Marked Redistribution Phase, as there is always data filled into every equivalence class, since there is no maximum cost constraint.

### Privacy Properties

The equivalence classes generated in Equivalence Class Creation fulfill *k*-anonymity by design. Each equivalence class is filled until it meets the chosen *k*. *t*-closeness is calculated after the anonymization process is complete, thus, the *t*-closeness constraint is to be checked afterward.

In the following we deduce the properties of *k*-anonymity and *t*-closeness being fulfilled by our algorithm. The set-values to our algorithm are the required *k*-anonymity and the bucket tree depth *b* which finally determines the *t*-closeness. We cluster our records in buckets. For these buckets we establish an upper bound for the error of a single representative for the bucket to all elements included. This error allows us to have an over-approximation of the *t*-closeness during the creation of our equivalence classes. Fulfilling the *k*-anonymity is assured by filling sufficient records into an equivalence class. The resulting *t* of the found distribution will most likely be smaller and hence fulfill the requirements.

Let there be a database *S*, consisting of the recorded waves, which is used for the generation of a Bucket tree *BT* and an Equivalence class size tree *ET*, which is created using the Database *S* and the set of Buckets *B* selected from *BT* using the parameter bucket tree depth *b*. *ET* provides a set of equivalence classes *EQC*, where *eqc*_*i*_ ∈ *EQC* are its members. One can overapproximate the *t* by adding up the maximum errors, like shown in^12^ for each member of every bucket in the equivalence class. Let *err*_*i*_ be the error associated with the bucket *b*_*i*_ and the representative of the bucket *r*_*i*_. Let’s denote a member of *b*_*i*_ as *r*_*j*_ ∈ *b*_*i*_ and the distance between two members as a function $dist({r}_{1},{r}_{2})\mapsto {{\mathbb{R}}}^{\ge 0}$.

$$er{r}_{i}=\mathop{\max }\limits_{1\le j\le | {b}_{i}| }\{dist({r}_{j}\in {b}_{i},{r}_{i})\}$$

I.e., the error of a bucket is the maximum distance between the representative of the bucket *r*_*i*_ to the members with the biggest distance to this representative. The overall error of an equivalence class can be denoted as

$$err(eq{c}_{i})=\mathop{\sum }\limits_{j=1}^{| eq{c}_{i}| }\{er{r}_{j}\cdot {p}_{j}\}\ge {t}_{i}$$

where *p*_*j*_ is the proportionality coefficient for this bucket, as was used by^12^. The proportionality coefficient reflects the share of a single bucket to the whole database. This approach to sum up the buckets errors, while still obeying the proportionality constraint by^12^, yields again an overapproximation of the resulting equivalence class.

#### Algorithm 1

Prepare Anonymization.

#### Algorithm 2

Anonymization Overview.

#### Algorithm 3

Data Distribution Phase.

#### Algorithm 4

Unmarked Redistribution Phase.

#### Algorithm 5

Marked Redistribution Phases (Constrained/Relaxed).

This error *err*(*eqc*_*i*_) is the overapproximation of the achievable *t* for this equivalence class. Thus, the target *t*, for the set of equivalence classes which will be released can safely be set to

$$t=\mathop{\max }\limits_{1\le i\le n}({t}_{i})$$

All equivalence classes created using the proposed process fulfill *k*-anonymity by design, as long as there are enough members in the database to be able to assign at least *k* members to each equivalence class. Otherwise, no releasable equivalence class is produced. I.e., if the database is too small, our algorithm can not successfully distribute the records in the equivalence classes, such that the requirements are fulfilled. The target *t* is chosen implicitly via the bucket tree depth *b*, which is always achieved for all equivalence classes filled up successfully to the target size through the usage of the Equivalence size trees^12^. In Results we give some resulting *t* values for chosen *b* values. However, further research on the influence of the bucket tree depth *b* on the *t*-closeness, with respect to the specific application and the base of large datasets and specific questions that are researched on, is needed.

If the resulting *t* from the chosen Bucket tree depth is not sufficient, a number of actions may be performed. For example, a new distribution with different sensitivity may be calculated and used, or some unfitting data may be removed from the dataset. Finally, a convex hull over all curves in one equivalence class is computed as a representative. This hull is additive, i.e., two equivalence classes can easily be merged without much computational effort. Depending on the underlying data, an optimal information loss creates a narrow hull which in turn can still show the features of interest of the original curves without showing any individual curve.

In general *k*-anonymity addresses linking attacks for *k* ≥ 3. As for *k* = 1 the privacy is trivially violated, for *k* = 2 the two borders of the equivalence class leak the precise value of all members, only the actual linkage of the victim to one of the two candidates need to be performed, thus leading to a high risk. For any larger *k*, still only the two borders are known precisely, thus obscuring the precise value of all other members of the equivalence class and therefore giving an attacker few values to work with. A small risk of linking attacks based on the borders remain, as the individuals which make up parts of the hull. Since the hull can consist of changing individual’s values, this risk is further reduced in our application. The privacy is ensured by removing explicit identifiers and generalizing all quasi identifiers, thus reducing the re-identification of a person in a *k*-anonymized equivalence class to a guess with the chance at most $\frac{1}{k}$. The probability might be even worse for the attacker, depending on the generalization and resolution of the domain.

A second possible attack vector are the borders of a *k*-anonymized equivalence class. An attacker knows, that the borders are actual data points, thus the best guess for an attacker, as this reduces the domain-resolution specific chance of guessing the right value back to $\frac{1}{k}$. This also holds for the approach of this paper, as an attacker cannot reconstruct an individual curve from the given equivalence class. It is even harder as an attacker would have to make the correct guess *n* times in a row, where *n* is the number of data-points in the curve. Thus, the chance of guessing the correct curve is $\frac{1}{k\cdot n}$. Additionally, the resolution of the lower and upper bound being used to construct the hull does not have to be the same as the one of the target curves. Thus, further increasing the complexity for an attacker.

However, even if the attacker does not guess the correct value, the hull still leaks precise information about an individual. This was the motivation for the implementation of reconstruction by Nergiz *et al*.^15^, to avoid the usage of a perturbation for those cases. For the proposed approach of this paper reconstruction could be implemented, but is optional, as an attacker does not know if and when the curve which is the upper or lower hull changes.

In addition to *k*-anonymity, the employment of *t*-closeness protects against a distribution attack. It ensures the information gain an attacker can obtain about an individual from the data compared to the population is reduced. According to Li *et al*.^2^ this prevents distribution, similarity, homogeneity, and background-knowledge attacks for a sufficiently small *t*.

A curve-specific security threat arises from alignment issues. If an equivalence class is *k*-anonymized and one wave stops earlier, for the remaining time only (*k* − 1) waves are left to anonymize. Thus, this equivalence class becomes (*k* − 1)-anonymized for some time. This issue can occur both at the start and end of the data. The implementation of this paper prevents this issue, by holding the last value of a curve until the last value of the longest curve and the first value of the shortest curve at the start respectively.

Another known attack remains: data republication. If a researcher repeatedly publishes a changing set of data, with insertions, deletions, and updates of data entries or using the same data with different parameters e.g., for different publications with varying research questions, *k*-anonymity and *l*-diversity are not sufficient according to Xiao and Tao^25^. Presumably *t*-closeness is also no protection, as it is designed for a static dataset. Thus, for republication of datasets the proposed approach is not protected against reconstruction of the data entries over multiple releases, unless it is extended by *m*-invariance according to Xiao and Tao^25^, which was not implemented in the approach of this paper.

Additionally, an attacker might try to invert the anonymization mechanism. I.e., gain insights into the raw data by partially or in the worst case fully reversing the anonymization mechanism. To reduce such a risk it is of high importance to thoroughly prepare the data for the anonymization as was described earlier, e.g., ensuring all curves have a sufficient length, which means they start and end at the same time or are cropped to the sufficient length. In the case of an inadequate data prepossessing with an early end or late start of some curves, this can lead to an equivalence class being locally not fulfilling *k*-anonymity. As mentioned above, one may be able to learn information about up to two individuals for each equivalence class, i.e., the borders of it. Those might change over time, but at which point is hard to tell for an attacker. As the assignment of the curves from the buckets into the equivalence class is done deterministically, an attacker might be able to reconstruct some information from this if the equivalence classes have overlap. No actual attack addressing this vector is known to us.

The mentioned attack vectors can be addressed by introducing a level of uncertainty, via a small randomness factor when choosing the elements of the equivalence class and adding noise directly to the measured data. However, this randomness will always increase the information loss and thus reduce the utility of the data and can conflict with the physiological dynamics which are subject of the researched question. Hence, it should be subject of future research.

To summarize, to our knowledge there is no full procedure inversion possible for this mechanism and the risk of a partial inversion can be reduced by correctly preparing the data for the anonymization and refraining the republication of an existing dataset for a second research question without obeying additional measures like *m*-invariance. Further improvements can be implemented by adding noise and oversampling to the borders of the equivalence classes and introducing randomness into the data selection process for the equivalence classes.

## Results

All data presented in this paper is recorded from animal trials approved by the Dutch central commission for animal experiments (AVD10700202010347)^16^. Three datasets of curves were evaluated. Each curve is a fragment of a recording from one animal and each curve can be interpolated. There is Airway Flow (AWF) with 12 curves of roughly 400 data points each in eight seconds per curve (50 Hz resolution), Airway Pressure (AWP), with 28 curves of roughly 200 data points each during four seconds per curve (50 Hz resolution) and the Peripheral Oxygen Saturation (SPO_2_), measured via plethysmography with 12 curves with roughly 200 data points during 400 seconds per curve. For SPO_2_ the sampling rate is not constant and varies from 0.25-1 Hz, but has an 0.5 Hz resolution on average. All datasets were cut to the same length and shifted to the same time frame to eliminate any alignment issues. Obvious errors in the data like a measurement beyond the possible range of the sensor or out of the range of any physiological limit, e.g., where the sensor was disconnected, or pressures which would not be bearable by the lung should be removed, whenever it was possible to clearly identify the erroneous data as such. Typically, the removed data points are a drop to zero for a few measurements, with similar values before and after the drop. However, for all datasets in this evaluation, the data was chosen such that no data points had to be removed. For the split evaluation of AWF and AWP the datasets were divided the Windowed-Fréchet approach. AWF used a Window-size of 667 ms and the step size 334 ms. In the case of AWP the window-size was 340 ms and the step size 170 ms. It resulted it 13 segments for AWF and 12 for AWP. For SPO_2_ the data was split into 16 segments with a 25 s fixed-length split, though some variation in length might occur due to variations in the sampling rate of the recording.

For the evaluation of the developed approach, it is not feasible to evaluate all possible parameter combinations. Thus, a default configuration was chosen and a single parameter, e.g. *k*, was changed for every run. As a default *k* = 3 was chosen, as any lower value would result in an information loss of zero, as the upper and lower bound precisely represents the anonymized data, and it is the lowest value, which achieves anonymity. The default balancing factor of the Bucket Tree was set to *f* = 0.4, as with *f* > 0.5 the constraint might be unfulfillable, but a small *f* can lead to a shallow Bucket Tree, which is not able to represent complex distributions well. As a default hull resolution 50 ms was chosen for AWF and AWP and 500 ms for SPO_2_. The distribution sensitivity *s*_*d*_ is the threshold for the generation of the population distribution. It should not be too small, as this will result in many small clusters, which can lead to a large *t* or few equivalence classes, as either not all equivalence classes can have members of every cluster, or few equivalence classes can be created. For the unsplit datasets it was 4000 for AWF and AWP, SPO_2_ used a *s*_*d*_ of 200,000. The split datasets used a *s*_*d*_ of 200 for both AWF and AWP, SPO_2_ used a *s*_*d*_ of 9000. For the single breath datasets *s*_*d*_ was set to 70 for both the heterogeneous and the homogeneous dataset. This leads to the default value for the Bucket Tree depth of *depth*_*b*_ = 1, as it is the lowest sensible depth. The impact of the drop threshold *d* and the equivalence class sensitivity *s*_*eqc*_ was not evaluated. They were set to *d* = *s*_*eqc*_ = 1.0, thus neither dropping nor splitting occurs. Per default all data is taken greedily from the buckets.

The presented parameter variation was done to evaluate resulting changes in computation time and information loss. For future evaluations the selection of parameters should be further researched. Bayesian optimization or Sequential Model-Based Optimization are promising, well researched methods to tune the parameters for expensive to evaluate functions like the proposed anonymization mechanism.

The evaluation of the duration, i.e., time to calculate the results, was performed on an AMD Ryzen 5 PRO 4650U, 2100 MHz, 6 Cores and 16 GB DDR4 RAM. In order to reduce the impact of random noise to the duration, e.g., differences in scheduling or background tasks (e.g., garbage collection) of the non-realtime capable operating system, the evaluation was repeated ten times. The implementation used for this evaluation did not employ parallelization of tasks, which could be used in future releases.

**Fig. 3:**
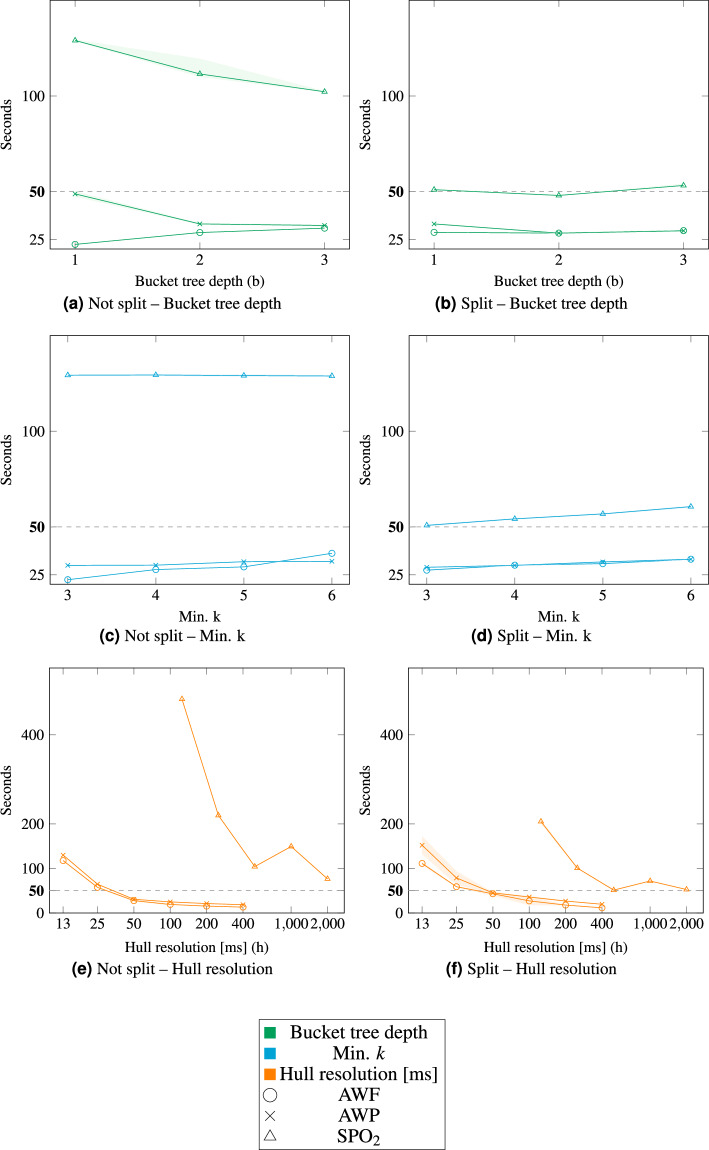
Duration of the anonymization for different parameter sets (*n* = 10).

**Fig. 4:**
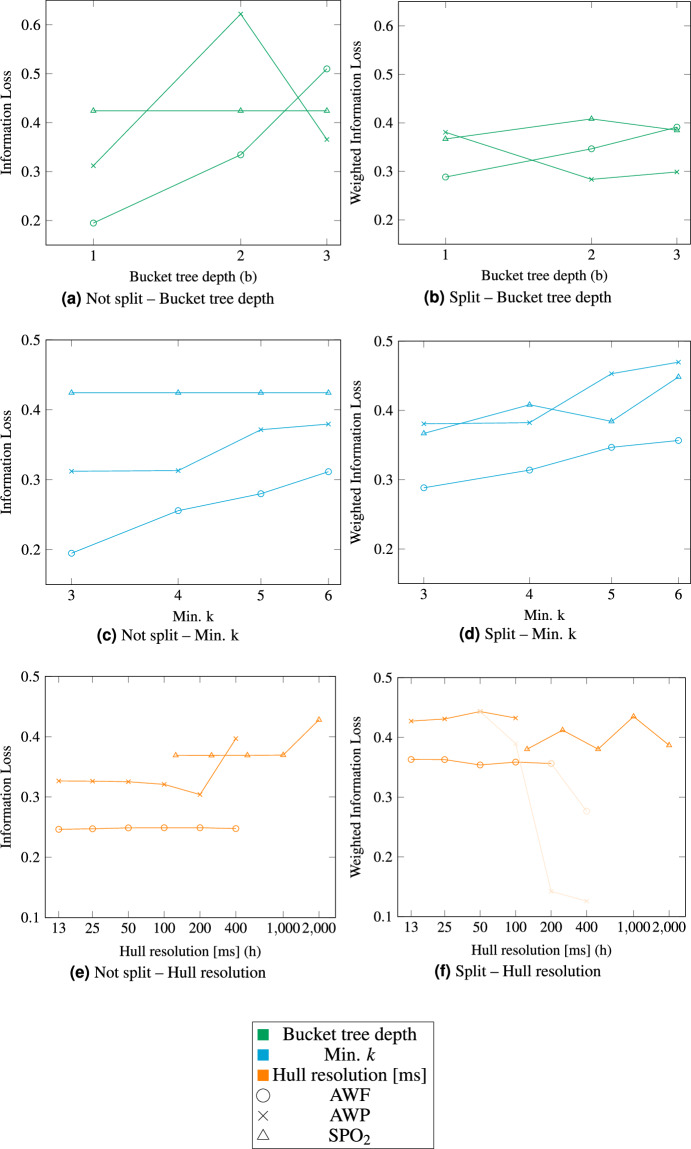
Information Loss of the anonymization for different parameter sets.

When looking at Figs. 3 and 4, the impact of the semi-local approach becomes apparent. Figure 3 shows the duration of anonymization for different parameter sets. Each set was repeated ten times, resulting in a 95 %-confidence band, with an assumed normal distribution, for the duration, which is drawn slightly transparent around the mean. For both figures it is hardly visible for most parameters, as the deviation is too small. In Fig. 3a,c,e the duration for SPO_2_ is about two times as high as in Fig. 3b,d,f. The largest impact on the duration has the resolution of the hull. For the other datasets, the effect is much smaller, this is due to the low number of data points in each curve. When the step size of the hull is decreased, i.e., the sampling rate is increased, the duration increases exponentially. The Bucket Tree depth has only a slight impact on the duration, if any. The minimal *k* constraint has a varying effect on the duration, both for the evaluation performed on the data which was split into segments along the time axis and for the evaluation performed on the whole data without splitting. If the duration increases or decreases with the minimal *k* thus depends on the underlying data. In any case the effect is rather small compared to the effect of the hull resolution on the duration.

As the algorithm is deterministically, the variation in runtime is due to the scheduling of the operating system and will still lead to the same results, given the same data and configuration.

The mean of the standard deviation for all runs of the parameter values of hull resolution can be seen in Table 1.

**Table 1:** Duration standard deviation.

*⌀SD*	AWF		AWP		SPO_2_	
	not split	split	not split	split	not split	split
H	87 ms	2.1 s	115 ms	6.1 s	325 ms	826 ms
K	48 ms	85 ms	75 ms	429 ms	256 ms	270 ms
B	43 ms	233 ms	44 ms	562 ms	612 ms	138 ms

In addition to the duration, the information loss was evaluated in the same setting in Fig. 4. An information loss of 0 means that no information is lost in the process of anonymization, whereas a loss of 1 indicates a complete loss of all usable information. As the assignment is done deterministically, there is no confidence band or mean standard deviation.

The assessment of the information loss for the split and unsplit cases should be comparable. Therefore, we also determine the information losses for a batch of split datasets for the entire extent of the original dataset. However, as there are initially many individual information losses, these must be combined to arrive at a single value. We want to avoid a very short segment having as much influence on the resulting single value as a much longer segment. We have therefore decided to weight the individual segments in the case of the split dataset according to their temporal proportion of the entire dataset.

The split data in Fig. 4b,d,f performs similar to the not split data in Fig. 4a,c,e, w.r.t. the information loss. For the split datasets the Information loss seems to decrease (the lighter orange curve) with a more course hull resolution, this is due to segments being smaller than the chosen resolution. This effect disappears when omitting all segments shorter than the chosen hull resolution. The original results are shown slightly transparent in such a case, whereas the results without the omitted segments are drawn normally. If the segments, which would be omitted make up more than 25 % of the curves, no cleaned result is shown. For AWP this happens for a hull resolution of 100 ms, where one segment of a length of 60 ms is omitted, the data points for 200 ms and greater are not cleaned, as the segments, which would be omitted would make up 30 % and 86 % of the curve. For AWF the no data point is cleaned, because only the one at 400 ms hull resolution would be worth cleaning, but the omitted segments would make up 29 % of the data. SPO_2_ does not have any data points which had to be cleaned. As SPO_2_ was split using the fixed approach, which does not take the underlying structure of the data into account, a worse result w.r.t. information loss would have been expected. However, comparing the information losses of a low number of different datasets, with varying split strategies is not sufficient to draw any meaningful conclusions. Thus, further research into the effects of different split strategies on the results would be needed.

The hull resolution does not have an impact on the information loss, whereas it is increased by the Bucket Tree depth. This must be seen somewhat in perspective, as the default distribution clustering resulted in few clusters. A low Bucket Tree depth, with few buckets would be suitable. For a different distribution clustering the result might be the other way around. As to be expected the minimal *k* constraint increases the information loss. This is the case, as for most datasets, an equivalence class with more members most likely increases the width of the equivalence class, thus increasing the information loss.

**Fig. 5:**
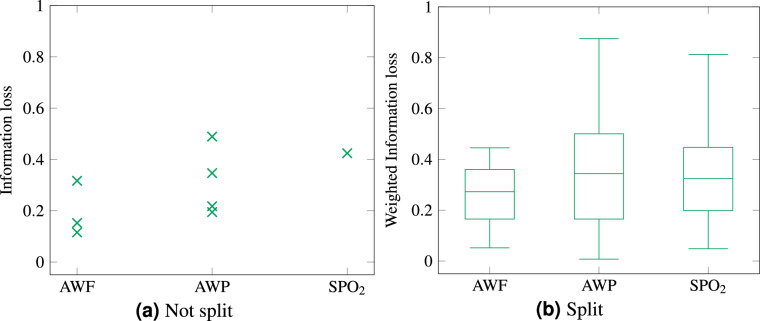
Information loss of the anonymization – box plot (*k* = 3).

The average information loss for the equivalence classes is evaluated with more details for *k* = 3 in Fig. 5. In Fig. 5a,b the information loss is shown. In this configuration the not split evaluation resulted in at most four equivalence classes per dataset, whereas the not split case has 20 to 30 equivalence classes in multiple segments. Therefore, Fig. 5a is given as a scatter plot.

The median information loss is much slightly lower or similar for the split dataset, compared to the not split dataset. With a median around 0.27 to 0.34 for all split datasets and one of 0.15 to 0.42 for the not split datasets.

For the airway flow we also calculated the closeness *t* explicitly for the three different bucket tree depths *b* = 1, which results in a *t*_*mean*_ = 0.440854769; *b* = 2, which results in a *t*_*mean*_ = 0.409989231 and *b* = 2, which results in a *t*_*mean*_ = 0.434829. We recognize the necessity to further explore the impact of *b* on the resulting *t* using larger datasets, particularly in relation to specific research questions being addressed.

**Fig. 6:**
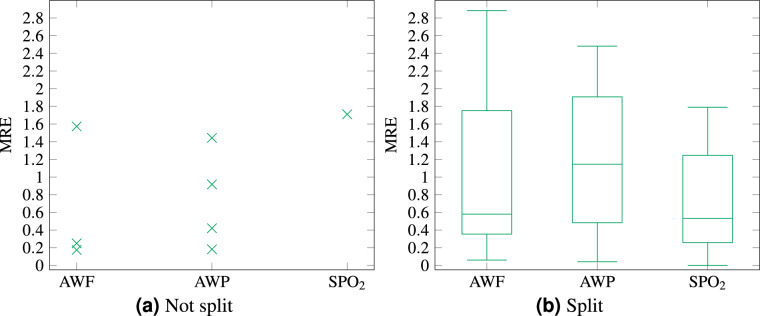
Median Relative Error of the anonymization – box plot (*k* = 3).

In Fig. 6 the Median Relative Error (MRE) is evaluated. The relative error is the deviation of the members from the hull, i.e., the distance to the upper or lower bound, depending on which one is closer. The MRE is the median of all relative errors in the equivalence class. It is calculated using the same resolution as the hull; thus, the data of the member curves is interpolated where the hull data-point does not match the member curve data-point. If interpolation is disabled for the dataset, the last, or closest, depending on the configuration of the framework, data point is used for the calculation whenever a value for a given timestamp is needed, but at this precise timestamp no data was recorded.

The deviation of the MRE is much smaller in Fig. 6a, compared to Fig. 6b. The not split evaluation resulted less than five equivalence classes, whilst the not split case has twenty to thirty equivalence classes in multiple segments. Therefore, Fig. 6a is given as a scatter plot, while Fig. 6b is a box plot. However, the median of the MRE is smaller for the long dataset of SPO_2_ using the split dataset, but larger for the other short datasets with the low resolution. In the split datasets it is for AWF  ≈ 0.58, for AWP  ≈ 1.14 and for SPO_2_  ≈ 0.53. In the unsplit datasets it is for AWF  ≈ 0.25, for AWP  ≈ 0.42 and for SPO_2_  ≈ 1.71. Additionally, some equivalence classes have a much smaller MRE for all parameters in Fig. 6b, compared to Fig. 6a.

**Fig. 7:**
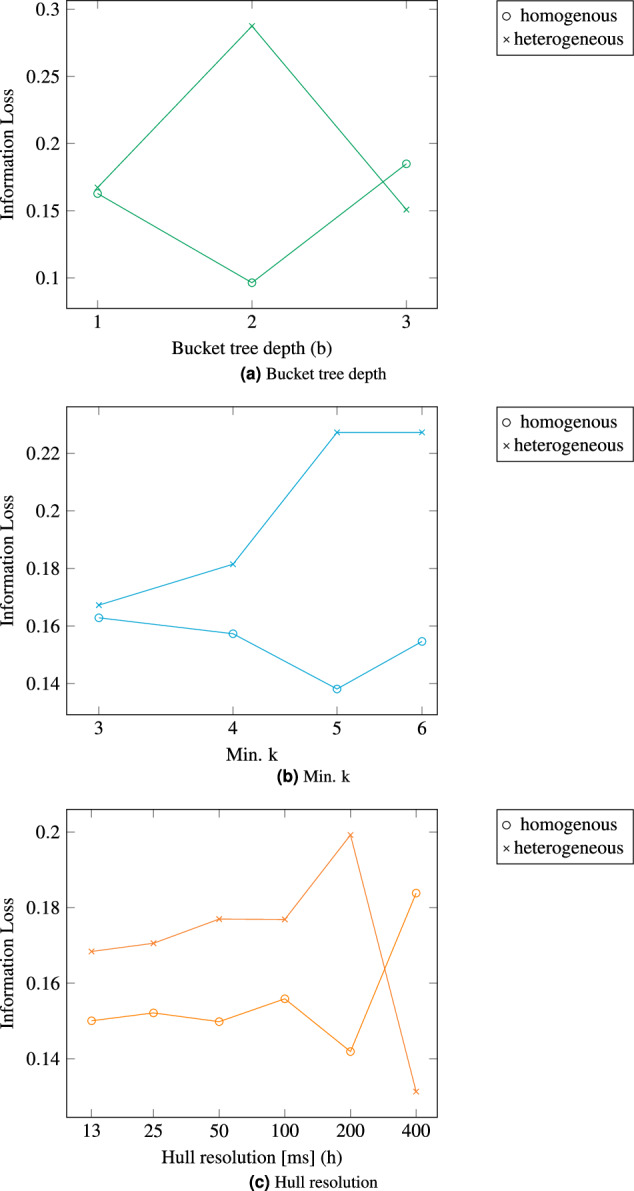
Information Loss of the anonymization of individual breath for different parameter sets.

For the split dataset AWP, visualized in Fig. 7, there are equivalence classes with a MRE of up to 2.2. Those are always in the small segments with the single cluster distributions. This can happen if the distribution does not match the underlying data, e.g., if some segments contain very diverse data, whereas other segments are comprised of homogeneous data. Thus, different distribution sensitivities would be needed for those segments. Additionally, it can be noted, that the achieved *t*, was always smaller for the split datasets.

Up to this point no physiological knowledge about the used datasets was used. The clustering of similar curves addresses the conservation of the signal’s dynamics. However, one can expect an influence if physiological knowledge is used. Hence, two further datasets were created by extracting airway flow (AWF) of single breaths^16^. The classification of single breaths reflects the physiological knowledge. One consists of homogenous and one of heterogeneous data, respectively. The homogenous dataset consists of 150 randomly picked curves. Each containing a single breath with a breathing frequency (RR) of 50. Those were picked from 1534 available curves of five animals. The heterogeneous dataset consists of 150 randomly picked curves, each containing a single breath with an RR between 15 and 70. Those were picked from 8754 available curves, with ten different RR-values of eight animals. The *s*_*d*_ was set to 70 for both datasets. The Information loss of both datasets with varying parameters is depicted in Fig. 7.

**Fig. 8:**
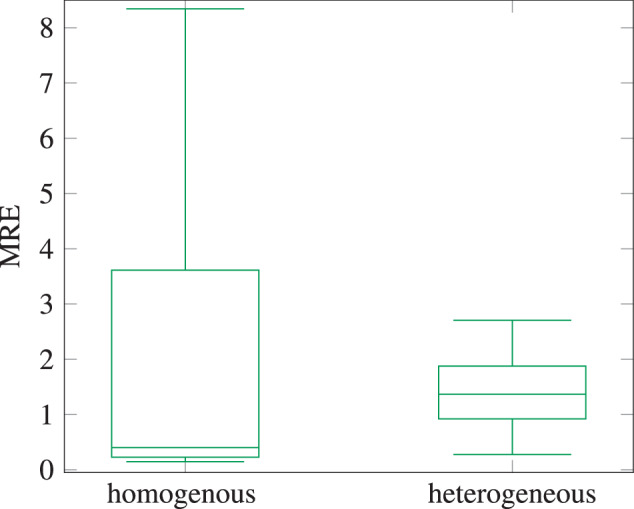
Median Relative Error of the anonymization of individual breath – box plot (*k* = 3).

For both datasets in Fig. 7 the Information loss showed no clear connection with the Bucket Tree depth, this is in contrast to the previous results in Fig. 4. Those results can be seen in Fig. 7a. However, it only shows that the Bucket Tree depth must be chosen with the distribution and structure of the underlying data in mind and there is no optimal set of parameters, which fits every dataset and distribution. For the minimal *k* in Fig. 7b, the first data point (*k* = 3) is the worst for the homogeneous dataset and decreases with a growing *k*, until there is a slight increase for *k* = 6. In the case of the heterogeneous dataset the first (*k* = 3) is the best parameter and the information loss increases with a growing *k*, until a plateau seems to be reached with *k* = 6. Like in the datasets before, the Hull resolution, which can be seen in Fig. 7c, has only a slight impact on the information loss. In Fig. 8 the MRE of the two single breath datasets are evaluated. It shows a low median for both boxplots, but some equivalence classes with very high median relative errors. This is comparable to the results from the other datasets.

## Discussion

A method to anonymize time-continuous personal data was needed in order to improve the privacy of participants, when releasing the data of a medical study. In order to achieve this goal, the methods for anonymizing data need to be extended to handle continuous data where the spatio-temporal relation is important. As the truth of the data should be maintained, a generalizing approach is chosen. This is *k*-anonymity, as introduced by Sweeney^1^. As it does not protect against distribution attacks, *t*-closeness is introduced by Li *et al*.^2^.

It is inefficient, both with respect to runtime and information loss, to enforce *t*-closeness after the data was already *k*-anonymized. Thus, a *t*-closeness centered approach similar to SABRE by Cao *et al*.^12^ is proposed. As the existing approaches^9,12,15^ do not maintain the spatio-temporal relation, or are not well suited for a *t*-closeness centered approach, a new one was proposed. It is bucketization based like SABRE by Cao *et al*.^12^, uses the Fréchet distance, as described by Alt and Godau^17^ and Eiter and Mannilla^18^, as a similarity metric. For the *t*-closeness the EMD^13^ is used to quantify the distances between distributions. As a real population distribution is hardly available for most time-continuous types of data, Distribution Clustering is introduced in this paper to generate distributions based on available data. In order to decrease the runtime and increase the local behavior of the algorithm the data is split, thus performing only semi-local decisions. The Windowed-Fréchet mode was developed in this paper to split the data with respect to the similarity and thus producing a variable length segmentation, partitioning the data. To allow an easy adaption of the data and an implicit selection of the maximum cost, Bucket Trees are introduced in this paper. They are used together with the Equivalence Class Size Trees, which provide the target sizes for the distribution of the data.

In order to anonymize the data, while maintaining *t*-closeness and achieving *k*-anonymity, a multiphase algorithm to (re)distribute data into equivalence classes is introduced in this paper. This reduces the number of dropped data entries, while still achieving good results with respect to the information loss.

The split dataset performs better for data with long, or high density curves and is faster, without sacrificing security. The developed approach achieves good results with respect to the information loss and median relative error, if the parameters are chosen right^16^. All while maintaining a low runtime and providing good protection against linking and distribution attacks by achieving a low *t*, which was always lower than the set target for *t*. While choosing the maximum cost and *t* parameters explicitly, many configurations were observed, where after a long calculation no valid solution could be found. With the introduction of Bucket Trees, the implied maximum cost and implied *t*, there were no longer any parameter configurations observed, where the constraints could not be met. Thus, always leading to a valid solution.

The reference value of a cluster is chosen randomly as the first value added to the cluster and not changed afterward, in order to save on runtime, as calculating new representatives within each cluster would mean to calculate pairwise Fréchet-distances between all values in the cluster. Other algorithms for finding the reference values shall be explored in the future.

A future progression could introduce a more context aware, which is physiology reflecting, splitting technique, which takes application specific requirements into account and does not solely rely on general trajectory-based heuristics. Additionally, the algorithm could be tested on further data, especially on different types of data. Furthermore, the algorithm could be extended, to allow a more connected approach to anonymization. I.e., link together multiple data types of a patient, e.g., some discrete data and some trajectories and anonymize them in such a way, that this grouping, especially the cross data type correlations are preserved.

With the current implementation, curves with a high cost are dropped segment-wise. Thus, in one segment the curve might be used, in the next it might be dropped from the anonymization. This could be improved by keeping track of the dropped curve segments and the curves they belong to and only dropping a curve if a majority of its segments were dropped.

As the metrics used in the proposed approach fulfill the triangulation equation, an approximate version of the metrics could be used^12,15^, similar to SABRE-AK. E.g., a mapping of the curve to Hilbert-space for the classification could potentially save computational time. Additionally, different distribution modes, like a case-centered mode, which creates an equivalence class around a given set of curves could be implemented. Furthermore, reconstruction of the equivalence classes, similar to^15^ would increase the security of the approach even further. However, the random reconstruction of Nergiz *et al*.^15^ is not suitable for the time-sensitive data. Thus, a different approach like weighted random reconstruction or random reconstruction within the confidence intervals of the equivalence class would be needed, to make large jumps of the representative less likely.

Anonymization is often an act of balancing privacy and utility. To find such a balance is application- and data-specific and can rarely be done for all types of data and intended applications. In this paper this balance can be evaluated as the minimal size of the equivalence classes i.e., min *k* and the achieved information loss. With a higher *k* more privacy is achieved and with a lower information loss the utility of the data is higher. Thus Fig. 4c and d are mainly relevant for this balance. This shows a linear decrease of the utility, i.e., an increase of the information loss, with an increase of the privacy, i.e., min. *k*. However, one should keep in mind that the utility for an individual equivalence class may vary. As can be seen in Fig. 5. To strike this balance in general or even for a subset of this class of data further research is needed. Hence, we plan in future studies to evaluate the performance on larger datasets and subsequently evaluate the utility of the anonymized dataset with classification algorithms we are working on currently.

### Related Work

Different approaches to prepare clinical data for publication are reported. The defined goal is often to verify one’s algorithms performance on the locally existing dataset without disclosing personal data of patients or allow for re-identifications.

Neubauer and Heurix^26^ identified the need to preprocess medical data prior to publication in order to ensure the patients data security. They came up with an encryption based protocol to handle medical data. This results in the need for a dedicated infrastructure to serve the different stakeholders in the process. The stakeholders comprise authorized healthcare professionals, the data owners, who are the patients, and affiliated persons, who can be relatives. Based on this access protection to the data a pseudonymized dataset can be generated. This approach also offered an anonymized secondary use by splitting the meta-information from the actual data. This unfortunately will also eliminate all information on the course of a data stream, which drastically lowers the utility of the data (e.g., losing the timestamp of a measurement). Linking attacks were not discussed in this work.

Majeed and Lee^27^ published a review on “Anonymization Techniques for Privacy Preserving Data Publishing”, which identifies hospital data as well as bank, social network, and insurance data to need for anonymization prior to publishment. They were able to derive the need for measures like *k*-anonymity, *l*-diversity, and *t*-closeness, especially for medical data^28^. Here, the aspect of imbalanced datasets anonymization is identified as an open research question which needs further elaboration than solely enforcing a specified *t*-closeness level.

Olatunji *et al*.^29^ identified in a review a distinction between different types of health data. Namely: (i) relational and (ii) graph-based. These types focus on the patient-cohort view. **Relational data** classifies single patients’ measurements in a single hospital where **graph-based data** addresses inter-patient or inter-hospital relationships. Again, the need for *k*-anonymity, *l*-diversity, and *t*-closeness is identified in this review. However, they use the rather low-time-resolution MIMIC-III dataset, which has aggregated measurements over a minute-wise timespan, with regularly one data point per hour. For example, blood pressure is given as systolic, diastolic or mean pressure but not as the continuous measurement waveform of the pressure sensor. Especially, if research questions focus on periodical physiological processes like heart-strokes or breaths, which comprises single events, the preservation of the sensors’ dynamics is crucial.

Guillaudeux *et al*.^30^ present a method that implements a local model, which allows for the generation of new random synthetic data, which is called “avatar data”. This method supports multivariate datasets. Their worked example is based on cancer data. If this approach is carried on to time-continuous data, we either need to have according models for the physiological and pathological processes the data is based on, or we will lose the according dynamics in the time-series. Again, this loss of dynamics will inhibit methods handling periodic high frequency physiological processes.

As upcoming usage of artificial intelligence (AI) brought up the approach to use neural networks, often specially generative adversarial networks (GANs), to generate anonymized data based on the trained AI methods^31,32^, we also want to discuss these. Generally speaking these stochastic methods are not deterministic. The Authors claim to preserve the same level of privacy as differential privacy by generating GAN based data. However, when considering the process of training GANs it becomes obvious that an according huge amount of labeled and pre-filtered data is needed for the process. Additionally, it can become legally hard to join datasets from different hospitals or even countries in order to overcome bias factors.

Differential privacy was identified as one possible measure to defend against attacks on anonymized data^29,31^. Hence, we want to differentiate more in detail to this method in the following.

#### Differential Privacy

As introduced, the application of differential privacy (DP) is an approach to address the risk of inference in patient related medical data. Generally speaking, DP algorithms need fairly large datasets and are initially developed for individual measurements and not time series data. When considering clinical data, we need to address time series data and explicitly need to conserve the dynamics of each individual monitored vital sign of the patient. Additionally, depending on the disease of interest, the affected number of patients and hence the available datasets are rather small.

There are applications of DP on time series data, however these adaptions need to incorporate application domain knowledge to enable the preserving of the needed dynamics^33^. We see the option to also incorporate domain knowledge in our proposed algorithm, but do not want to implement this as a compulsory need. The first step in this direction is the clustering of comparable sequences to buckets.

Since the available data basis in our application (mechanical ventilation on neonatal intensive care unit) is quite small we came up with the described new method and did not evaluate an adaption of DP to our data.

## Data Availability

There are two general types of data, the first contains possible multiple physiological cycles per wave, e.g., breaths, the second contains only one breath per wave. All data was acquired during in-vivo experiments^16^, which were reviewed and approved by Netherlands National Committee for the protection of animals used for scientific purposes (NCad) (AVD10700202010347). The first type of data contains three datasets of curves: AWF, AWP and SPO_2_, they are evaluated in Figs. 3 to 6. The second type only evaluates AWF in two datasets, which are randomly picked out of a large pool of available curves. They are evaluated in Figs. 7 and 8. One set consists of only curves with a respiratory rate of 50 per minute, i.e., a homogeneous dataset, the other is heterogeneous and thus consists of curves with a respiratory rate in the range of 15 to 70 per minute. All data is stored as a CSV, the value delimiter is the semicolon. Before the actual data a header is added. First the UUID which identifies the patient in our database, then a start date in ISO-Datetime format. The third line is the header for the date, i.e., what value it is and in square brackets its unit. The first column will always be the time in seconds, the second column will be the value of the curve. The files of unsplit data of the first type is just enumerated and named after the dataset. The split data of this type appends a segment identification to each file so that all segments of one curve can be identified. The files of the second type of data start with an indication of the respiratory rate, followed by a short identifier of the patient. Afterward the type of the dataset (AWF) follows, next is the type of breath, which was identified by an algorithm of the group. At the end of each file a numerical identifier is incremented, it is reset to one for each patient. The two datasets of the second type, with 150 curves each share the same filename structure as the ones above, except they have the start date of the breath as an identifier after the patient identifier instead of the enumeration at the end. All resulting data being calculated out of the presented physiological data introduced above using the presented algorithms can be found at https://git.rwth-aachen.de/informatik11/medical-data-anonymization/-/blob/Evaluation-Results/VentilationData.zip.

## Code availability

The code used for this publication is publicly available at: https://git.rwth-aachen.de/informatik11/medical-data-anonymization^34^. The tool was created using Qt5. The variable *s*_*d*_ “sensitivity of distribution” from this paper is labeled *v* in the software and *s*_*e**q**c*_ “sensitivity of equivalence class” is labeled *s* in the software. Both variables are renamed in the algorithms of this paper for better readability.
